# Tetramethylpyrazine Ameliorates Peritoneal Angiogenesis by Regulating VEGF/Hippo/YAP Signaling

**DOI:** 10.3389/fphar.2021.649581

**Published:** 2021-04-13

**Authors:** Xiaolin Zhu, Yun Shan, Manshu Yu, Jun Shi, Lei Tang, Huimin Cao, Meixiao Sheng

**Affiliations:** ^1^ Department of Nephrology, Affiliated Hospital of Nanjing University of Chinese Medicine, Nanjing, China; ^2^ Department of Nephrology, The Second Affiliated Hospital of Nanjing University of Chinese Medicine, Nanjing, China

**Keywords:** peritoneal fibrosis, peritoneal angiogenesis, peritoneal vascular endothelial cells, YAP, Golgi, tetramethylpyrazine

## Abstract

Angiogenesis of human peritoneal vascular endothelial cells (HPVECs), linked to vascular endothelial growth factor (VEGF)/VEGF receptor 2 (VEGFR2) signaling, is a complication of peritoneal fibrosis (PF). Hippo/YAP signaling interacts with VEGF/VEGFR2 signaling, but the effect on peritoneal angiogenesis and PF has not been studied. We tested VEGF/Hippo/YAP inhibition by tetramethylpyrazine (TMP) in PF mice and HPVECs. This treatment ameliorated peritoneal dialysis (PD)–induced angiogenesis and PF. In mice, PF was associated with upregulation of VEGF, and TMP ameliorated submesothelial fibrosis, perivascular bleeding, and Collagen I abundance. In HPVECs, angiogenesis occurred due to human peritoneal mesothelial cells (HPMCs)–conditioned medium, and TMP alleviated HPVECs migration, tube formation, and YAP nuclear translocation. YAP knockdown PF mouse and HPVEC models were established to further confirm our finding. YAP deletion attenuated the PD-induced or VEGF-induced increase in angiogenesis and PF. The amount of CYR61 and CTGF was significantly less in the YAP knockdown group. To study the possibility that TMP could benefit angiogenesis, we measured the HPVECs migration and tube formation and found that both were sharply increased in YAP overexpression; TMP treatment partly abolished these increases. As well, the amount of VEGFR localized in the trans-Golgi network was lower by double immunofluorescence; VEGFR and its downstream signaling pathways including p-ERK, p-P38, and p-Akt were more in HPVECs with YAP overexpression. Overall, TMP treatment ameliorated angiogenesis, PF, and peritoneum injury. These changes were accompanied by inhibition of VEGF/Hippo/YAP.

## Introduction

Peritoneal dialysis (PD) is a cost-effective, high-quality renal replacement therapy. One of the main limitations to successful long-term use of the method is the deleterious effects of peritoneal dialysis solutions (PDS) on the peritoneal membrane (PM). The peritoneum is composed of a monolayer of mesothelial cells, supported by connective tissue, underneath of which there are blood vessels, nerves, and lymphatic vessels. In long-term PD, the PM serves as a semipermeable barrier for ultrafiltration, and diffusion may present structural alterations, including peritoneal fibrosis (PF), vasculopathy, and angiogenesis, which are leading causes of peritoneal ultrafiltration failure (Roumeliotis et al., 2020).

Angiogenesis is an extremely complex process and usually defined as the formation of new blood vessels, controlled by a delicate balance of stimulatory and inhibitory growth factors. It is crucial not only for growth, tissue injury, repair, and healing, but also contributes to the pathogenesis of different inflammatory, fibroproliferative, and ischemic diseases (Zhang et al., 2017). In recent years, peritoneal angiogenesis is taken for a key event during the development of peritoneal fibrosis. The degree of vascularization correlates with the area of fibrotic tissue, suggesting the involvement of angiogenesis in the progression of PF (Elpek, 2015). Inhibition of peritoneal angiogenesis can significantly attenuate PF, emerging angiogenesis as a promising therapeutic target (Li et al., 2018; Jing et al., 2020). Therefore, the identification of molecules that regulate peritoneal angiogenesis and, in turn, the understanding of how these molecules function during the angiogenic cascade are major challenges in the field of antiperitoneal fibrosis.

Vascular endothelial growth factor (VEGF) as an extremely potent proangiogenic factor possesses a dominant role in mediating endothelial cell (EC) sprouting, migration, and network formation. These vital functions of VEGF are mediated by VEGF receptor 2 (VEGFR2), usually called VEGFR, expressed on ECs (Melincovici et al., 2018). VEGF is reported to be dysregulated in patients with cancer and fibrotic diseases, and its implication in peritoneal angiogenesis during PD is an intense research subject. As a matter of fact, VEGF is expressed abundantly in human PD effluents and in a rat PD model (Xiao et al., 2014; Hao et al., 2019). Moreover, PDS can promote the production of VEGF by human peritoneal mesothelial cells (HPMCs) (Liu et al., 2013). Thus, we hypothesize that VEGF/VEGFR may mediate mesothelial–endothelial crosstalk and promote peritoneal angiogenesis during PD.

At the same time, VEGF-mediated signaling in angiogenesis has been studied intensely. When the VEGF signal transmits into transcriptional programs, it is found to associate with the Hippo pathway (Elaimy and Mercurio, 2018). The Hippo pathway is a highly conserved signaling pathway that restricts proliferation and controls organ size. It consists of a kinase cascade mediated by Mst, which phosphorylates and activates Lats (Meng et al., 2016). Activated Lats directly phosphorylates YAP, causing its cytoplasmic sequestration, degradation, and inactivation. Transcription coactivator YAP has been identified as a master regulator of cell behavior (Azad et al., 2019). In fact, during angiogenesis, VEGF/VEGFR2 signaling axis critically requires YAP activity to mediate angiogenesis (Kim et al., 2017). Inhibition of the Hippo pathway results in the activation of YAP; then, YAP shuffles from the cytosol to the nucleus where it interacts with different transcription factors to regulate a large group of target genes (Zanconato et al., 2016), including several proangiogenic genes, such as connective tissue growth factor (CTGF) and cysteine-rich angiogenic inducer 61 (CYR61) (Boopathy and Hong, 2019). Interestingly, VEGFR2 turnover at the plasma membrane is dependent upon the delivery of the newly synthesized pool of receptor *via* the Golgi complex (Tiwari et al., 2013), and the depletion of YAP can block Golgi trafficking (Wang et al., 2017). Prompted by these observations, we wondered whether mesenchymal-derived VEGF could activate the Hippo/YAP pathway of endothelial cells during peritoneal angiogenesis.

Tetramethylpyrazine (TMP) is an alkaloid monomer isolated from Chinese herbal medicine Conioselinum anthriscoides “Chuanxiong” (syn. *Ligusticum* chuanxiong Hort.). It is widely used in the clinical treatment of various diseases, such as coronary heart disease, diabetes, cancers, and liver injury (Zhao et al., 2016). Accordingly, researchers explored the pharmacological capabilities of TMP in multiple molecular targets, such as anti-inflammation, antioxidant, antiplatelet, and antiapoptosis (Qing et al., 2019). Recently, a series of studies have shown that TMP possessed an antiangiogenesis effect. For example, the administration of TMP could enhance the antitumor effect of paclitaxel by inhibiting tumor cell proliferation, invasion, and suppression of metastasis in ovarian cancer xenograft mouse models (Zou et al., 2019). Additionally, treatment of Human umbilical vein endothelial cells (HUVECs) with TMP resulted in a significant inhibition of cell proliferation, migration, tube formation, and the expression of angiogenesis-related proteins (Yuan et al., 2018). In contrast, much less is known about the role of TMP in peritoneal angiogenesis.

In this study, we intended to determine the antiangiogenesis effect of TMP in a PD mouse model and in human peritoneal vascular endothelial cells (HPVECs)–HPMCs coculture system. Furthermore, we constructed YAP knockdown PF mouse and HPVEC models respectively by recombinant adeno-associated virus (AAV) and small interfering RNA (siRNA), as well as YAP overexpression HPVEC model by plasmid vector to dissect the molecular mechanisms of TMP in peritoneal angiogenesis.

## Materials and Methods

### Materials

TMP was obtained from Harbin Sanlian Pharmaceutical Co., Ltd. (Harbin, China). Vascular endothelial growth factor (VEGF) was bought from R&D Systems (Minneapolis, MN, United States). Standard PD solution (PDS) (Dianeal PD-2 peritoneal dialysis solution with 4.25% dextrose, pH 5.2) was supplied by Baxter Healthcare (Deerfield, IL, Unites States). Verteporfin was purchased from Absin Bioscience Inc. (Shanghai, China). AAV YAP and plasmid YAP were obtained from GeneChem Co., Ltd. (Shanghai, China). Lipofectamine 2000 transfection reagent was supplied by Invitrogen (CA, Unites States). Small interfering RNA YAP and riboFECTTM CP transfection reagent were purchased from RiboBio Co., Ltd. (Guangzhou, China). Matrigel matrix was bought from Corning Inc. (NY, Unites States). VEGF ELISA Kit was obtained from Jin Yibai Biological Technology Co., Ltd. (Nanjing, China). RPMI 1640, fetal bovine serum (FBS), and penicillin/streptomycin were supplied by Gibco (Thermo Fisher Scientific, Inc.). 4’, 6-Diamidino-2-phenylindole (DAPI), RIPA lysis buffer, phosphatase inhibitor, protease inhibitor, and crystal violet staining solution were purchased from Beyotime Biotechnology (Shanghai, China). Subcellular Protein Fractionation Kit for cultured cells and BCA Protein Assay Kit were supplied by Thermo Fisher Scientific (Waltham, MA, Unites States). The TRIzol reagent was purchased from Life Technologies (Gaithersburg, MD, Unites States). 2 × ChamQ SYBR qPCR Master Mix and 5 × HiScript II qRt SuperMix were obtained from Vazyme (Nanjing, China). The primers were supplied by Sangon Biotech (Shanghai, China). The anti-phospho-VEGFR2 (Tyr1175), anti-phospho-Lats1 (Thr1079), anti-P38, anti-phospho-P38 (Thr180/Tyr182), anti-ERK, anti-phospho-ERK (Thr202/Tyr204), anti-Akt, anti-phospho-Akt (Ser473), anti–β-actin, anti-histone H3 antibodies, secondary horseradish peroxidase (HRP)–conjugated goat anti-rabbit and anti-mouse IgG, Alexa Fluor 488 anti-Rabbit IgG, and Alexa Fluor 594 anti-mouse IgG were obtained from Cell Signaling Technology (Boston, MA, Unites States). The anti-YAP1, anti-phospho-YAP1 (Ser127), anti-CD31, anti-TGN46, and anti-VEGFR2 antibodies were obtained from Abcam (Cambridge, United Kingdom). The anti-VEGF, anti-CTGF, and anti-CYR61 antibodies were obtained from Santa Cruz Biotechnology (Texas, Unites States). The anti-Lats1 antibody was obtained from Proteintech Group (Wuhan, China). The anti-Collagen I antibody was obtained from Servicebio Technology Co., Ltd. (Wuhan, China). The ECL system was obtained from Millipore (Bedford, Unites States).

### Cell Culture and Stimulation

HPMCs (HMrSV5) were supplied by Jennio Biotech Co., Ltd. (Guangzhou, China), and HPVECs were purchased from Chengyuan Biotech Co., Ltd. (Shanghai, China). They were cultured in RPMI 1640 supplemented with 10% FBS (v/v) and 1% penicillin/streptomycin (v/v) in a humidified atmosphere with 5% CO_2_ at 37°C. HPMCs were incubated with PDS at various concentrations (1.5, 2.5, 3, 3.5, and 4.25%) for 12–48 h to get HPMC-conditioned medium (PM-CM), and the cell medium without PDS stimulation was used as negative control (N-CM). HPVECs were starved overnight in serum-free medium before treatment with reagents such as PM-CM, N-CM, 50 ng/ml VEGF, 60 nM verteporfin, and TMP with the final concentration ranging from 10 to 40 μM. For analyzing the phosphorylation status of Akt, Erk, P38, Lats, YAP, and nuclear localized YAP, VEGF was added to starved HPVECs for 2 h unless otherwise mentioned. For checking CTGF and CYR61 protein expression, VEGF was added to starved cells for 24 h. For checking CTGF and CYR61 mRNA expression, VEGF was added to starved cells for 6 h. For analyzing the transport of VEGFR, VEGF was added to starved cells for 30 min.

### Cell Viability Assay

Cell viability was measured in a 96-well plate using a quantitative colorimetric assay. The cells were cultured in 96-well plates in triplicate with various treatments. A CCK-8 solution (10 µL) was applied to each well, and all wells were incubated for another 1 h at 37°C. Absorbance was measured at 450 nm.

### Cell Proliferation Assay

HPVECs were seeded into a 96-well plate at a density of 5 × 10^3^ cells per well in complete media and cultured for 24 h for cell attachment. Cells were then starved overnight to achieve a quiescent state. After starvation, cells were treated with various treatments, such as PM-CM, TMP, VEGF, and verteporfin for 48 h. Cell proliferation was detected by CCK-8 assay.

### Wound Healing Assay

HPVECs were seeded in 6-well plates and incubated at 37°C for 24 h and then starved overnight. Confluent cells were scratched with a pipette tip. Subsequently, cells were induced by PM-CM and treated with TMP or verteporfin, either alone or in combination, for 12 h. Images were obtained before drug intervention and 12 h after drug intervention. Migration was quantified as the difference between the two cell-free wound areas. All assays were repeated three times independently.

### Transwell Migration Assay

Twenty-four-well transwell chambers with 8 µm pores were used. A HPVEC suspension containing 5 × 10^5^ cells/ml was seeded into the upper chamber with serum-free medium. The medium in the lower chamber contained 20% FBS (v/v). Cells were incubated at 37°C for 24 h. Cells on the upper surface of the membrane were removed using cotton swabs. Inserts were fixed with 4% paraformaldehyde for 15 min and then stained with crystal violet staining solution for 30 min.

### Tube Formation Assay

Matrigel matrix was thawed at 4°C overnight. A 96-well plate was coated with 10 μL matrigel matrix and incubated at 37°C for 30 min for polymerization. HPVECs were then resuspended in 100 μL medium containing treatment reagents, respectively, and seeded onto the matrigel-coated plate. After 6 h of incubation, tube formation was quantified by counting the number of total lengths in three randomly selected fields of view.

### Immunofluorescence Assay

The HPVECs were cultured in a 24-well plate and treated with reagents. After treatment, cells were fixed, permeabilized, and blocked. Next, cells were incubated overnight with the primary antibody at 4°C, followed by a fluorescent secondary antibody for 1 h at room temperature and staining with DAPI for 5 min. Images were acquired with a Zeiss AX10 fluorescence microscope (Carl Zeiss, Oberkochen, Germany).

Total RNA was extracted using TRIzol reagent according to the manufacturer’s instructions. RNA concentration and purity were measured with a spectrophotometer, and the target 260 nm/280 nm ratio was between 1.8 and 2.0. Reverse transcription was performed using a HiScript Q RT SuperMix Kit according to the manufacturer’s instructions. RNA expression was measured with a ChamQ SYBR qPCR Master Mix Kit and an ABI7500 real-time PCR system (Applied Biosystems, Foster City, CA, United States), according to the manufacturer’s instructions. β-Actin was used as an internal reference. The PCR human primers were designed as follows: CTGF: 5′-ATG​GGC​GAG​CTT​TGC​ACA​GAC-3′, 5′-GAC​TCC​TAT​GCG​GCG​GTT​GAT​G-3′; CYR61: 5′-TGC​TGC​GAG​GAG​TGG​GTC​TG-3′, 5′-CCT​CCC​TTC​ACG​ATG​GCA​ATC​AG-3′; and VEGF: 5′-GCC​TTG​CCT​TGC​TGC​TCT​ACC-3′, 5′-CTT​CGT​GAT​GAT​TCT​GCC​CTC​CTC-3′. The PCR mouse primers were designed as follows: CTGF: 5′-AGC​GGT​GAG​TCC​TTC​CA-3′, 5′-AGG​CAG​CTT​GAC​CCT​TCT-3′; and CYR61: 5′-TCT​GGA​GGC​ACC​CAA​GT-3′, 5′-GTC​GCA​GGG​CTG​AGT​TT-3′. All reactions were performed in triplicate, and the results were analyzed using the 2^-ΔΔCT^ method. The experiment was repeated at least 3 times.

### Western Blotting

The total proteins from mice peritoneum, HMrSV5, and HPVECs were extracted using ice-cold RIPA buffer containing phosphatase inhibitor and protease inhibitor cocktail. Nuclear, cytoplasmic, and cytomembrane proteins were obtained by Subcellular Protein Fractionation Kit. The BCA protein assay was used to detect the protein concentration. Electrophoresis and western blotting analysis were performed as described previously (Zhang et al., 2015).

### Cell Transient Transfection

HPVECs were transfected with 100 nM siRNA YAP using riboFECTTM CP transfection reagent and 0.1 g YAP plasmid using Lipofectamine 2000 transfection reagent according to the manufacturer’s instructions for 48 h. Then, cells were stimulated as indicated with treatment reagents.

### PF Mouse Model, YAP Gene Transfection in Mice, and Treatment with Reagents

Male C57BL/6 mice (20–25 g in weight) were obtained from Qinglongshan Animal Farm (Nanjing, China). The PF mouse model was developed by daily intraperitoneal injection with 4.25% dextrose PDS at a dose of 10 ml/kg/day for 30 days. Mice in the TMP groups were subjected to intraperitoneal injection with TMP diluted in PDS at the concentration of 40 and 80 nM. The YAP knockdown mouse model was established by injecting YAP recombinant AAV once into the tail vein at a dose of 1.00 E + 11 v. g/ml for 21 days before intraperitoneal injection of PDS. Under the guidance of the Animal Care and Use Committee, all of the animals were treated humanely and were randomly divided into six groups as follows: 1) control (*n* = 5); 2) peritoneal dialysis (injected intraperitoneally with PDS, *n* = 5); 3) low TMP treatment (injected intraperitoneally with PDS + 40 nM TMP, *n* = 5); 4) high TMP treatment (injected intraperitoneally with PDS + 80 nM TMP, *n* = 5); 5) YAP knockdown (YAP recombinant AAV, *n* = 5); and 6) YAP knockdown + peritoneal dialysis (YAP recombinant AAV + PDS, *n* = 5). After treatment, the abdomens of the mice were opened with a midline incision. Next, parietal peritoneum and subpyloric omentum samples that were 3 mm × 1 mm × 1 mm in size were removed for further examination.

### Histology and Immunohistochemistry (IHC)

Parietal peritoneum samples were fixed in 10% neutral-buffered formalin for 24 h, dehydrated successively in a graded alcohol series (75, 85, 95, and 100%, v/v), and embedded in paraffin. The samples were sectioned at a 5 μm thickness. For histological examination, the sections were stained with hematoxylin-eosin (HE) and Masson’s trichrome to quantify the pathological condition and peritoneum thickness. For IHC, the sections were stained with anti-Collagen I and anti-CD31 antibodies and then incubated with the appropriate secondary antibody. Protein expression was detected with a DAB kit. Images were obtained using an Olympus BX45 inverted microscope. After CD31 immunohistochemical staining, the microscopic images were fixed at the position where there were most vessels with ×100 magnification. The pictures of blood vessels were taken in several random fields, and the number of CD31-positive cells was counted as microvessel density.

### Enzyme-Linked Immunosorbent Assay

The amounts of VEGF secreted in culture media or PD fluid from the mice were determined using human and mouse VEGF ELISA Kit, respectively, according to the manufacturer’s directions. For the assessment of VEGF concentration in cell culture media, the PM-CM was used. For the assessment of VEGF concentration in PD fluid, a syringe was used to draw intra-abdominal fluid when the abdomen was opened, making sure that PDS was injected intraperitoneally 1 h ago.

### Statistical Analysis

All data from at least three independent experiments were expressed as the mean ± standard error of the mean (SEM) and were analyzed using GraphPad Prism 6.05 software (GraphPad Software). Values of *p* < 0.05 were considered statistically significant.

## Results

### Peritoneum VEGF/VEGFR Signaling Is Activated During PD

**FIGURE 1:**
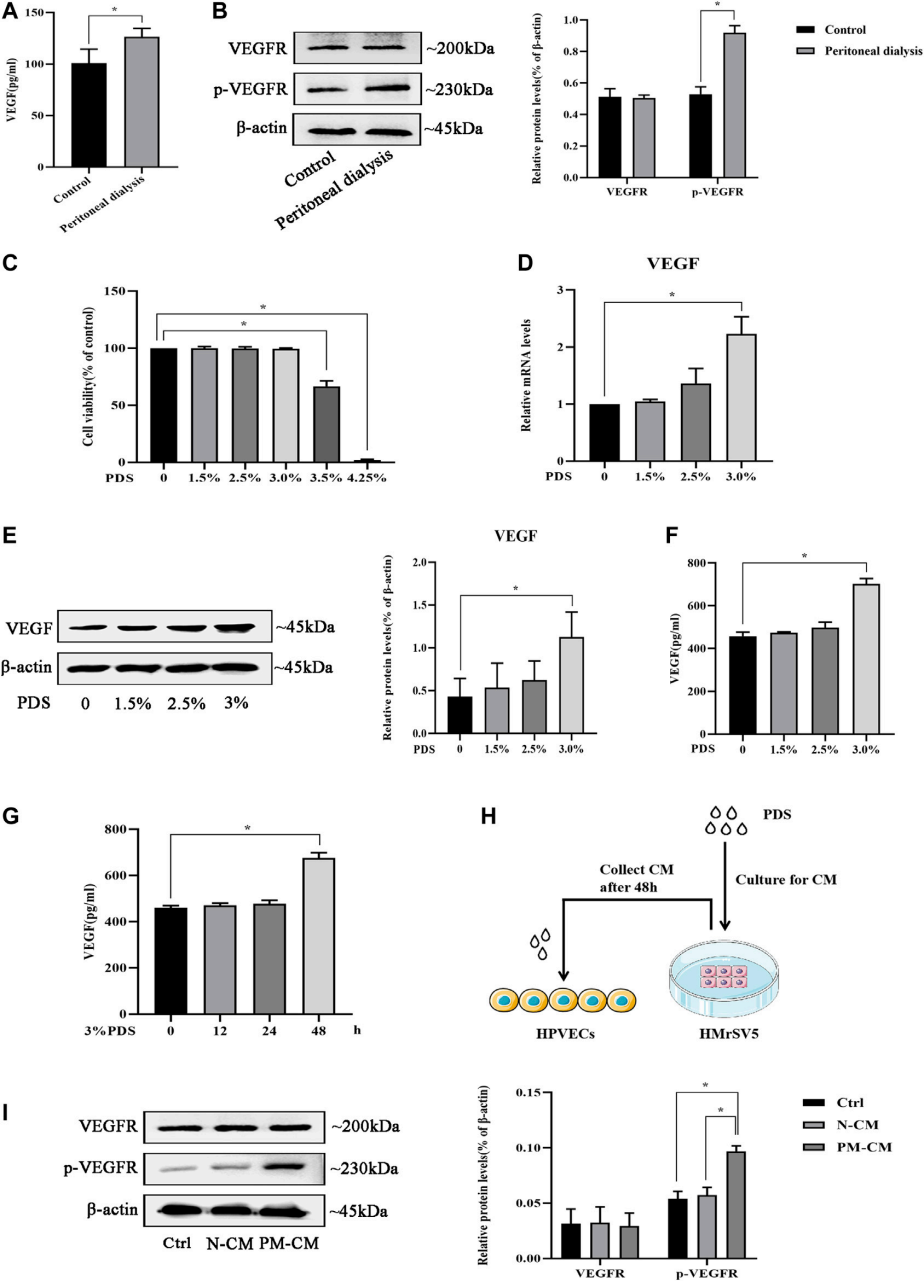
VEGF/VEGFR signaling is activated by peritoneal dialysis (PD). **(A, B)** PD mice were treated with 4.25% dextrose peritoneal dialysis solution (PDS) daily for 30 days. **(A)**VEGF concentrations were determined in the peritoneal lavage of the control group and the PD group by ELISA. **(B)** Western blotting assay was used to detect VEGFR and p-VEGFR protein expressions of peritoneal tissues in the control group and the PD group. **(C)** HMrSV5 cells were treated with PDS at various concentrations (0, 1.5, 2.5, 3, 3.5, and 4.25%). However, 3.5 and 4.25% dextrose PDS caused strengthened cell apoptosis. **(D, E)** HMrSV5 cells were treated with PDS at various concentrations (0, 1.5, 2.5, and 3%) and subjected to real-time PCR for VEGF mRNA expression **(D)** and western blotting for VEGF protein expression **(E)**. **(F, G)** ELISA was used to detect VEGF concentrations in HMrSV5 supernatants. **(F)** HMrSV5 cells were treated with serial concentrations of PDS (0, 1.5, 2.5, and 3%). **(G)** HMrSV5 cells were treated with 3% dextrose PDS at different times (0, 12, 24, and 48 h). **(H)** Pattern diagram of HPVECs cocultured with HMrSV5. **(I)** VEGFR and p-VEGFR protein expressions were assessed by western blotting in HPVECs cocultured with HMrSV5. Ctrl: control; PM-CM: HPMC-conditioned medium; N-CM: negative control of conditioned medium. Data represent mean ± SEM of at least 3 independent experiments (*p < 0.05).

Angiogenesis is confirmed to involve in the progression of peritoneal fibrosis, and VEGF is a potential proangiogenic factor; we further investigate whether there is VEGF-induced peritoneal angiogenesis during PD. First, we detected VEGF concentration in PD fluid *in vivo*. Compared with the control group, PD fluid from mice injected with 4.25% dextrose PDS showed a significant increase in VEGF concentration (Figure 1A). Moreover, the protein level of p-VEGFR in the parietal peritoneum in the PD group significantly increased with unchanged VEGFR (Figure 1B). To study the origin of VEGF in PD, we mimicked the *in vivo* conditions. HMrSV5 were exposed in PDS with various dextrose concentrations, 3.5 and 4.25% dextrose PDS induced loss of cellular viability, and 1.5, 2.5, and 3% dextrose PDS were nontoxic to cells (Figure 1C) which were used in the subsequent experiments. Then, we found that VEGF mRNA and protein levels increased in a concentration-dependent manner (Figures 1D,E). VEGF concentration in cell culture supernatants also showed an increase and reached a peak at the stimulation of 3% dextrose PDS for 48 h (Figures 1F,G). Thus, HMrSV5 culture supernatants stimulated with 0 and 3% dextrose PDS (separately called N-CM and PM-CM) for 48 h were used as a stimulant to coculture with HPVECs (Figure 1H). Compared with control, the PM-CM enhanced the protein level of p-VEGFR in HPVECs, with unchanged VEGFR (Figure 1I). These data showed that VEGF derived from HMrSV5 can activate HPVECs VEGF/VEGFR signaling during peritoneal dialysis.

### TMP Attenuates Peritoneal Angiogenesis in a Mouse Peritoneal Fibrosis Model of PD

**FIGURE 2:**
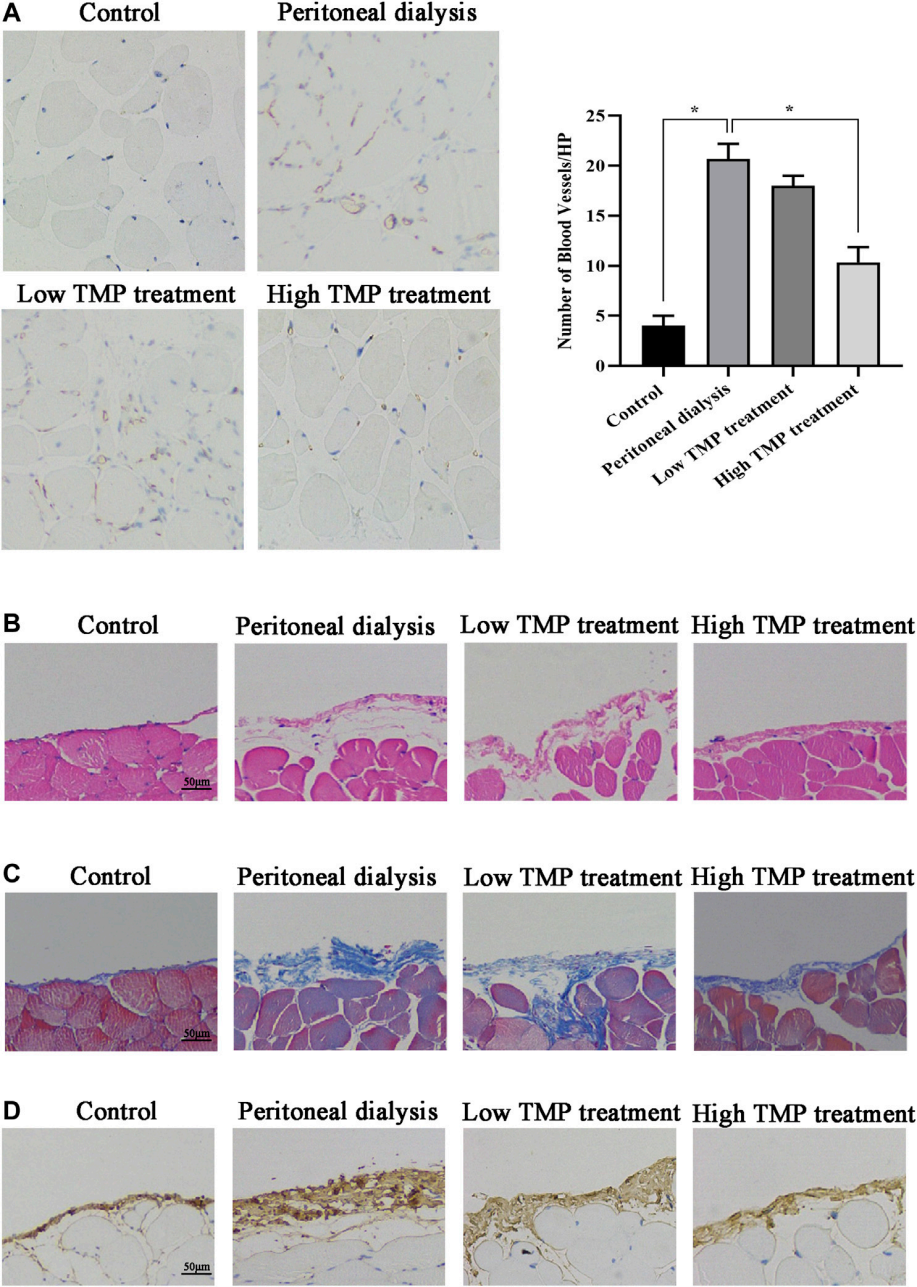
Effect of TMP on peritoneal angiogenesis and fibrosis in PD mouse. Mice were injected intraperitoneally with PDS, TMP diluted in PDS to form the final concentrations of 40 and 80 nM. **(A)** Representative fields showed the different vessel densities in the parietal peritoneum after immunohistochemistry staining with anticluster of differentiation 31 (CD31) (EC marker) (×100). The number of blood vessels per field after anti-CD31 staining, assessed in the peritoneum under ×100 magnification (graph). Data represent mean ± SEM (*p < 0.05). **(B)** HE staining of mesothelial cells of the parietal peritoneum in mice showed mesothelial injury and fibrosis (scale bar = 50 μm). **(C)** Peritoneal thickening and ECM deposition were observed using Masson’s trichrome staining (scale bar = 50 μm). **(D)** Tissues of the peritoneum were subjected to immunohistochemical staining for Collagen I expression (scale bar = 50 μm).

To observe whether peritoneal angiogenesis happened during PD, we counted the number of vessels in the peritoneum after CD31 (a marker of capillary sprouting) immunohistochemical staining. Compared with the control group, the number of CD31-positive cells increased significantly in the PD group. The microvessel density of the high TMP treatment group was markedly lower than that of the PD group (Figure 2A). At the same time, we observed whether the effect of TMP on peritoneal fibrosis was consistent with that on peritoneal angiogenesis. HE staining was performed to evaluate the histological changes in the peritoneum. In the PD group, HE staining showed peritoneum mesothelial layer denudation, inflammation, submesothelial fibrosis, and perivascular bleeding, while the high TMP treatment group exhibited significantly alleviated mesothelial injury and fibrosis of the submesothelial zone (Figure 2B). Masson staining showed that peritoneal tissue of the PD group markedly thickened with a rough surface, and the mesothelial cells were swelling and partly deficient with massive collagen accumulation and deposition. Compared with the PD group, all the abovementioned pathological changes and the peritoneal thickness obviously attenuated in the high TMP treatment group rather than the low TMP treatment group (Figure 2C). IHC analysis was performed to observe the expression of Collagen I which is a marker of fibrosis. Compared with the control group, Collagen I in the mesothelial and submesothelial areas significantly increased in the PD group, but these abnormal expressions were attenuated in the high TMP treatment group (Figure 2D). Thus, TMP with high concentration was chosen for the subsequent experiments *in vivo*. In the observation, we carefully observed that peritoneal angiogenesis has related to peritoneal fibrosis, and TMP could alleviate peritoneal angiogenesis as well as peritoneal fibrosis.

### TMP Inhibits Peritoneal Angiogenesis in HPVECs Cocultured with HMrSV5

**FIGURE 3:**
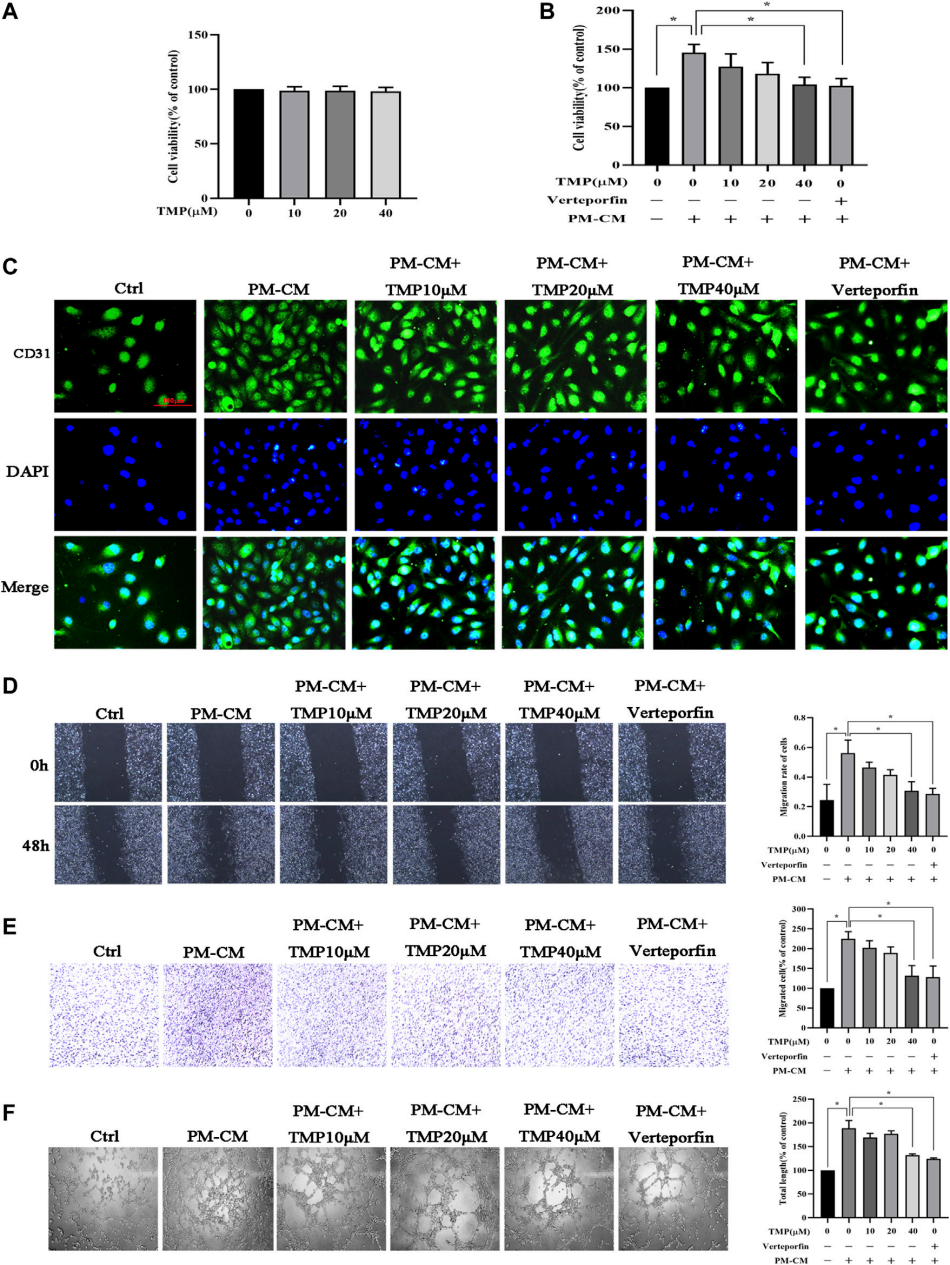
Effect of TMP on peritoneal angiogenesis *in vitro*. **(A)** HPVECs were treated with serial concentrations of TMP (0, 10, 20, and 40 μM) for 24 h. Neither strengthened cell proliferation nor apoptosis was observed. **(B–F)** HPVECs were exposed to PM-CM with or without various concentrations of TMP (0, 10, 20, and 40 μM) and 60 nM verteporfin. Cell proliferation was detected by CCK-8 assay **(B)** and immunofluorescence assay for visualizing endothelial cells marker (scale bar = 100 μm) **(C)**. Migration of HPVECs was detected by wound healing assay **(D)** and transwell migration assay **(E)**. **(F)** Tube formation was quantified by counting the total length in three randomly selected fields of view. Data represent mean ± SEM of at least 3 independent experiments (*p < 0.05).

To investigate the effect of TMP on peritoneal angiogenesis *in vitro*, firstly we studied the cytotoxicity of TMP using a CCK-8 assay. Treatment of HPVECs with up to 40 μM TMP for 48 h had almost no effect on cell viability (Figure 3A). Subsequently, we detected HPVECs proliferation also by CCK-8 assay. The sustained stimulation of PM-CM for 48 h induced a significant HPVECs proliferation, but 40 μM TMP effectively reduced the abnormal expression (Figure 3B). The changes could also be seen in the immunofluorescence of CD31 (a marker of endothelial cells) (Figure 3C). In addition, 40 μM TMP notably reversed HPVEC migration increased by PM-CM stimulation in wound healing assay (Figure 3D) and transwell migration assay (Figure 3E). Angiogenesis was also assessed using a tube formation assay. Quantitative measurements showed that PM-CM significantly increased the total length, which was remarkedly decreased in combination with 40 μM TMP (Figure 3F). Thus, 40 μM TMP was chosen for the subsequent experiments *in vitro*. Meanwhile, pharmacological inhibition of the Hippo pathway with 60 nM verteporfin achieved the same phenotype as 40 μM TMP (Figures 3B–F). Collectively, these results confirmed the antiperitoneal angiogenesis role of TMP *in vitro*, and at this point, the effect of TMP is similar to Hippo pathway inhibitor verteporfin.

### TMP Upregulates YAP Phosphorylation and Inhibits YAP Nuclear Translocation

**FIGURE 4:**
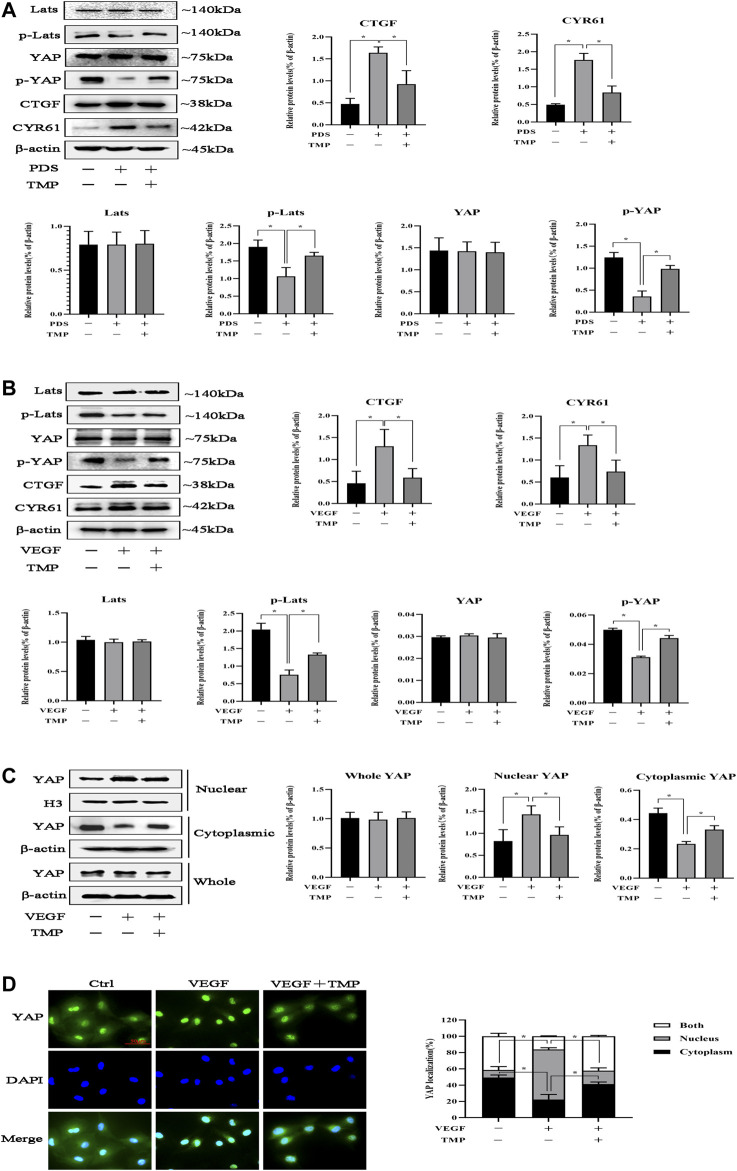
Effect of TMP on YAP nuclear translocation. **(A)** Mice were treated by PDS with or without 80 nM TMP. Peritoneal tissues were homogenized using RIPA buffer for western blotting assay. Protein expressions of Lats, p-Lats, YAP, p-YAP, CYR61, and CTGF were detected. **(B–D)** HPVECs were exposed to 50 ng/ml VEGF with or without 40 μM TMP. **(B)** Western blotting assays were used to detect Lats, p-Lats, YAP, p-YAP, CYR61, and CTGF of HPVECs. **(C)** HPVECs were lysed using the nuclear and cytoplasmic extraction reagents to obtain nuclear and cytoplasmic proteins. Western blotting assays were used to detect nuclear and cytoplasmic YAP expression. Histone H3 and β-actin were used as the loading controls for the nucleus and cytosol, respectively. **(D)** Immunofluorescence assays were performed to observe the subcellular translocation of YAP. YAP expressions in the cytoplasm and nucleus were visualized based on the green fluorescent signal. The images were obtained by fluorescence microscopy (scale bar = 50 μm). Data represent mean ± SEM of at least 3 independent experiments (*p < 0.05).

We next investigated the mechanisms of TMP inhibiting peritoneal angiogenesis. It had been proven that TMP attenuated peritoneal angiogenesis like verteporfin, and we considered that TMP might also have the ability to inhibit the Hippo pathway. As shown in Figure 4A, Hippo/YAP signaling was activated during PD, which was characterized by decreased phosphorylation of Lats and YAP. Compared with control, the protein expressions of CTGF and CYR61 were increased after PDS stimulation, while TMP distinctly reversed all these changes. To further examine the cellular mechanisms, 50 ng/ml VEGF was used as a trigger of HPVECs angiogenesis. We demonstrated that VEGF could also activate Hippo/YAP signaling. Compared with stimulation of VEGF alone, hypophosphorylation levels of Lats and YAP were upregulated and the protein expressions of CTGF and CYR61 were downregulated by the treatment of VEGF combination with TMP (Figure 4B). Indeed, YAP acts as a transcription factor to initiate downstream gene transcription. We next explored YAP nuclear translocation during peritoneal angiogenesis. At first, nuclear and cytoplasmic proteins were obtained, and western blotting revealed that the protein expression of YAP decreased in the cytoplasm but increased in the nucleus after VEGF stimulation, whereas it was not changed in the whole cell. TMP treatment reversed these abnormal expressions (Figure 4C). An immunofluorescence assay also demonstrated that staining of nuclear YAP was notably reduced by VEGF stimulation and significantly enhanced by TMP treatment (Figure 4D). Taken together, these data suggested that TMP upregulated YAP phosphorylation and inhibited YAP nuclear translocation as an initial step to attenuate peritoneal angiogenesis.

### YAP Is Involved in HPVEC Migration and Tube Formation

**FIGURE 5:**
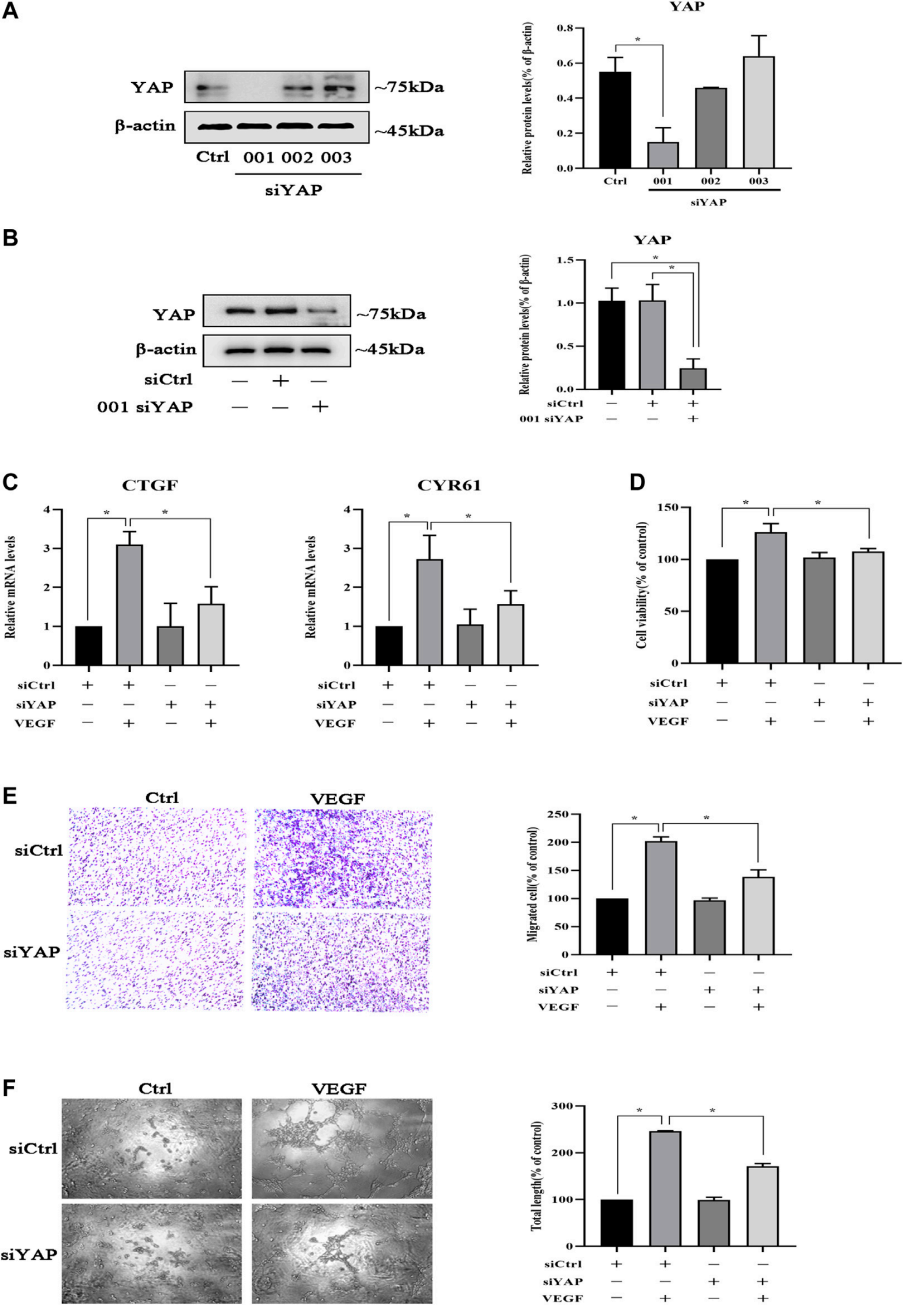
YAP is involved in the inhibition of HPVEC migration and tube formation. **(A)** HPVECs were transiently transfected with three kinds of SignalSilence YAP siRNA for 48 h. Then YAP protein expressions were analyzed by western blotting. **(B)** HPVECs were transiently transfected with the SignalSilence YAP control siRNA (siCtrl) and 001 siRNA for 48 h. Western blotting was also used to detect YAP protein expression. **(C–F)** HPVECs were treated with siCtrl or YAP siRNA for 48 h and incubated with VEGF (50 ng/ml). **(C)** CTGF and CYR61 mRNA levels were analyzed by real-time PCR. **(D)** Cell proliferation was detected by CCK-8 assay. **(E)** Migration of HPVECs was detected by transwell migration assay. **(F)** Tube formation was quantified by counting the total length in three randomly selected fields of view. Data represent mean ± SEM of at least 3 independent experiments (*p < 0.05).

Since TMP inhibited peritoneal angiogenesis by regulating YAP, we established YAP knockdown HPVECs to study the effect of YAP in peritoneal angiogenesis. As shown in Figure 5A, HPVECs received three kinds of siRNA specific for YAP to knockdown YAP; the 001 siRNA for YAP worked best and was chosen for the subsequent experiments. Then, HPVECs received either a negative control siRNA or 001 siRNA for YAP. The results showed that 001 siRNA could knockdown YAP, while negative control siRNA could not affect the expression of YAP protein (Figure 5B). After the YAP knockdown HPVECs were established, VEGF was given to induce HPVEC angiogenesis. CYR61 and CTGF mRNA levels were upregulated by VEGF stimulation, but this effect was partially reversed by the knockdown of YAP (Figure 5C). In addition, knockdown of YAP could inhibit VEGF-induced HPVEC proliferation, migration, and tube formation (Figures 5D–F). These results suggested that YAP played an important role in HPVEC proliferation, migration, and tube formation.

### YAP Is Implicated in Peritoneal Angiogenesis in Mouse Model

**FIGURE 6:**
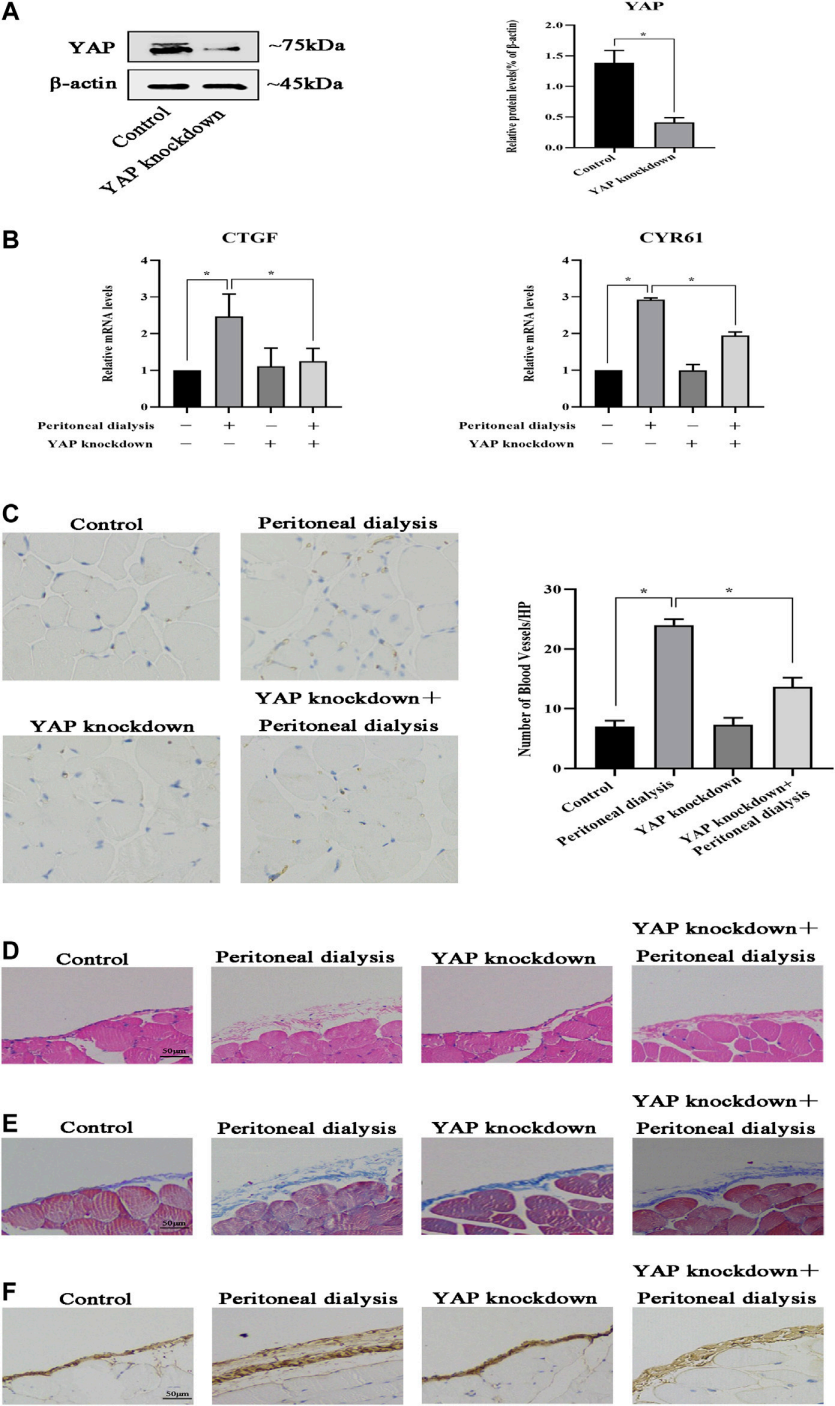
YAP is involved in peritoneal angiogenesis *in vivo*. Mice were injected intraperitoneally once with YAP recombinant AAV at a dose of 1.00E + 11 v.g/ml. After 21 days, the mice were injected intraperitoneally with PDS for another 30 days. Control: normal control; peritoneal dialysis: standard PDS (10 ml/kg/day); YAP knockdown: YAP recombinant AAV (1.00E + 11 v.g/mL); YAP knockdown + peritoneal dialysis: YAP recombinant AAV (1.00E + 11 v.g/mL) + standard PDS (10 ml/kg/day). **(A)** Western blotting was used to detect YAP protein expression of the parietal peritoneum in the control group and the YAP knockdown group. **(B)** CTGF and CYR61 mRNA levels in the parietal peritoneum were analyzed by real-time PCR assay. Data represent mean ± SEM (*p < 0.05). **(C)** Representative fields showed the different vessel densities in the parietal peritoneum after immunohistochemistry staining with anti-CD31 (×100). The number of blood vessels per field after anti-CD31 staining, assessed in the mesentery under ×100 magnification (graph). **(D)** HE staining of mesothelial cells of the parietal peritoneum in mice showed mesothelial injury and fibrosis (scale bar = 50 μm). **(E)** Peritoneal thickening and ECM deposition were observed using Masson’s trichrome staining (scale bar = 50 μm). **(F)** Tissues of the peritoneum were subjected to immunohistochemical staining for Collagen I expression (scale bar = 50 μm).

To further elucidate the effect of YAP on peritoneal angiogenesis, we generated the YAP gene transfection of mouse by AAV in an intraperitoneal injection with constructs inhibiting YAP expression. As shown in Figure 6A, compared with the control group, YAP protein expression was significantly decreased in the YAP knockdown group. CYR61 and CTGF mRNA levels were upregulated in the PD group, whereas this abnormal expression was partially reversed by YAP knockdown (Figure 6B). Then, we determined CD31 immunohistochemical staining and the microvessel density in the fibrotic mouse parietal peritoneum. Compared with control, the number of CD31-positive cells increased significantly in the PD group. However, YAP knockdown resulted in the decline of microvessel density compared with that in the PD group (Figure 6C). In addition, it had been previously demonstrated that peritoneal angiogenesis was associated with peritoneal fibrosis; then, we observed whether remission of peritoneal angiogenesis could ameliorate peritoneal fibrosis. HE staining demonstrated that YAP knockdown alleviated mesothelial injury and fibrosis of the submesothelial zone in PD mice (Figure 6D). Masson staining showed that YAP knockdown relieved the peritoneal thickness, massive collagen accumulation, and deposition in PD mice (Figure 6E). IHC analysis also performed that expression of Collagen I significantly increased in the PD group, whereas intraperitoneal injection by TMP with high concentration caused a decrease in the abnormal expression (Figure 6F). Altogether, these results suggested that YAP had a vital role in peritoneal angiogenesis, and peritoneal fibrosis could get alleviated when peritoneal angiogenesis was in remission.

### TMP Inhibits HPVEC Migration and Tube Formation by Targeting YAP

**FIGURE 7:**
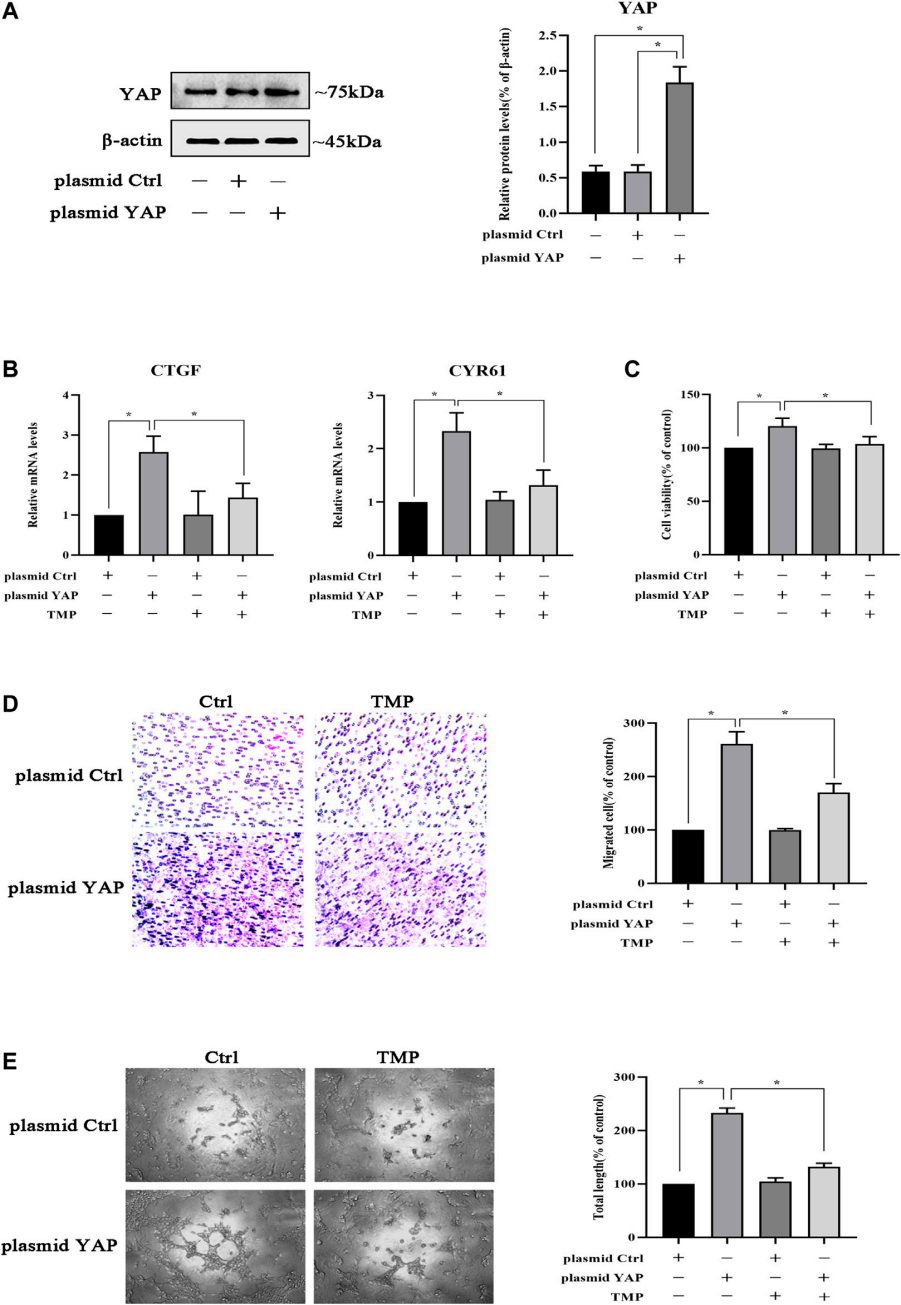
TMP inhibits HPVEC migration and tube formation by targeting YAP. **(A)** HPVECs were transiently transfected with the control plasmid and the overexpression YAP plasmid for 48 h. Then western blotting was used to detect YAP protein expression. **(B–E)** HPVECs were transiently transfected with the control plasmid and the overexpression YAP plasmid for 48 h and subsequently treated with TMP (40 μM). **(B)** HPVECs were lysed using the TRIzol reagent and then subjected to real-time PCR to measure mRNA levels of CTGF and CYR61. **(C)** Cell proliferation was detected by CCK-8 assay. **(D)** Migration of HPVECs was detected by transwell migration assay. **(E)** Tube formation was quantified by counting the total length in three randomly selected fields of view. Data are expressed as mean ± SEM (*p < 0.05).

Due to the importance of YAP in peritoneal angiogenesis, we further studied whether TMP directly acted on it. HPVECs were transfected with plasmid YAP to make YAP overexpression; meanwhile, the negative control plasmid did not affect YAP expression (Figure 7A). Moreover, the mRNA levels of CTGF and CYR61 were upregulated after YAP overexpression in HPVECs, while these abnormal high expression levels were alleviated by TMP treatment (Figure 7B). Furthermore, overexpression of YAP increased HPVEC proliferation, migration, and tube formation, but TMP effectively reversed the changes (Figures 7C–E). Taken together, these data suggested that YAP was used as a target in the effect of TMP inhibiting HPVEC proliferation, migration, and tube formation.

### TMP Impairs VEGFR Trafficking from Golgi to Cell Surface

**FIGURE 8:**
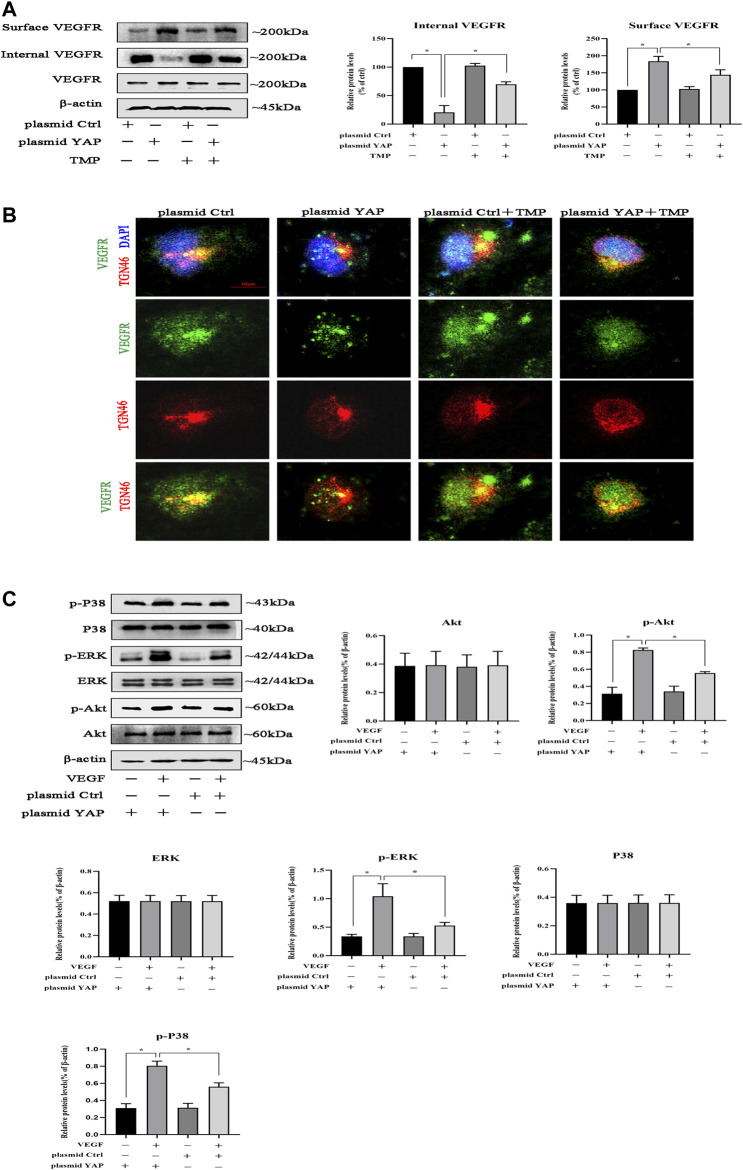
Effect of TMP on VEGFR trafficking from Golgi to the cell surface. HPVECs were transiently transfected with the control plasmid and the overexpression YAP plasmid for 48 h and subsequently treated with TMP (40 μM) or VEGF (50 ng/ml). **(A)** HPVECs were lysed using Subcellular Protein Fractionation Kit to obtain cytomembrane and intramembrane proteins. Western blotting assays were used to detect cell surface and internal VEGFR expression. **(B)** Immunofluorescence assays were performed to observe the VEGFR and TGN46 (trans-Golgi network [TGN] marker). Representative images of VEGFR localization in the TGN of the transfected HPVECs (scale bars = 10 μm). **(C)**) Representative blots for p-ERK, p-P38, and p-Akt were detected by western blotting assays, showing enhanced VEGFR downstream signaling in YAP overexpression cells. Data represent mean ± SEM (*p < 0.05).

Studies had showed that YAP played a crucial role in trafficking VEGFR from the trans-Golgi network (TGN) to the plasma membrane (Wang et al., 2017); we tested whether TMP could impair the transports of VEGFR by targeting YAP. Firstly, HPVECs were transfected with plasmid ctrl or plasmid and then treated with TMP. We obtained the cytomembrane and internal proteins by using Subcellular Protein Fractionation Kit. Western blotting performed showed that VEGFR protein was not changed in the whole cells, but increased in the surface and decreased in the internal when YAP was overexpressed in HPVECs; the abnormal expressions were reversed in a certain degree after TMP treatment (Figure 8A). Consistently, double immunofluorescence was used to observe the colocalization of VEGFR and TGN46 (a marker for trans-Golgi network [TGN]). Compared with control, the amount of VEGFR localized in the TGN46 was lower in HPVECs with YAP overexpression, but the change was reversed after TMP treatment (Figure 8B). We reasoned that if VEGFR of the cell surface increased after YAP overexpression, the activation of VEGFR and its downstream signaling pathways should also be increased. Consistent with this reasoning, after VEGF stimulation, the downstream signaling events of VEGFR, such as p-ERK, p-P38, and p-Akt, were increased much more in HPVECs with YAP overexpression (Figure 8C). Collectively, these data suggested that there was a positive feedback loop related with angiogenesis in HPVECs. TMP could establish a proper lower VEGFR trafficking process to inhibit the positive feedback during peritoneal angiogenesis.

## Discussion

**FIGURE 9:**
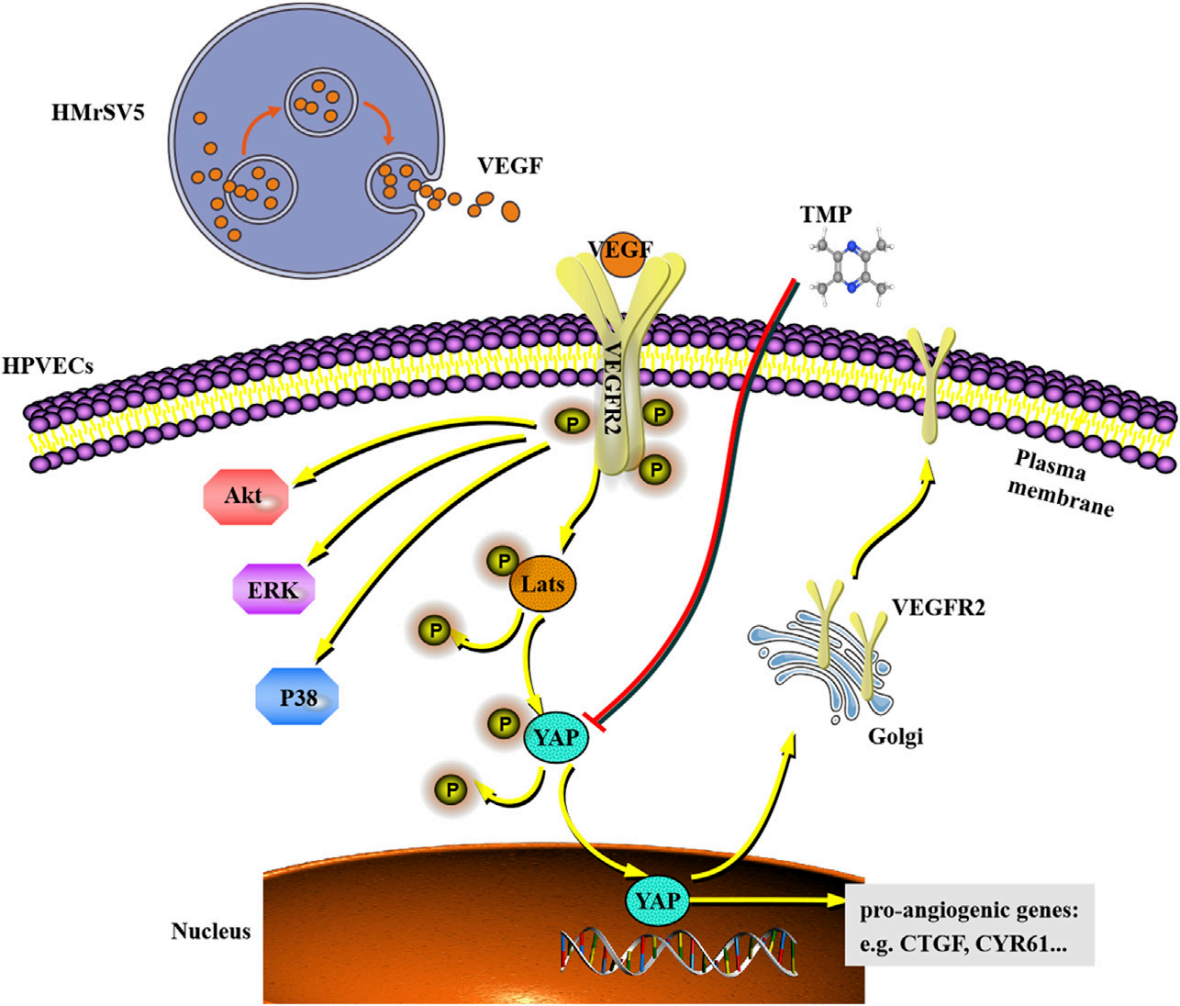
Working model shows that VEGF derived from HMrSV5 regulates the migration and tube formation of HPVECs via a mechanism which involves YAP nuclear translocation. As a small molecule inhibitor, TMP weakens YAP nuclear translocation and VEGFR trafficking induced by YAP, which may even further ameliorate activation of VEGFR downstream signaling.

In this study, we demonstrated that 1) TMP inhibits angiogenesis and fibrosis in a mouse model of PD; 2) TMP inhibits migration and tube formation in peritoneal vascular endothelial cells; 3) the beneficial effect of TMP on peritoneal angiogenesis is attributed to the inhibition of YAP nuclear translocation; and 4) TMP also weakens VEGFR trafficking from the trans-Golgi network to the plasma membrane, which may further ameliorate activation of VEGFR downstream signaling pathways. All of these results proved that TMP (PubChem CID 14296) is a small molecule inhibitor of the Hippo pathway and ameliorates peritoneal angiogenesis both *in vitro* and *in vivo*, which may even suppress peritoneal fibrosis (Figure 9).

Sustained exposure of high-glucose PD solution induces peritoneal fibrosis and triggers an increased angiogenesis. Morphologically, it is characterized by a detachment of the mesothelial layer, angiogenesis, a progressive thickening with ECM deposition, and an increased presence of myofibroblasts which were responsible for collagen production (Balzer, 2020). Peritoneal angiogenesis results in increased production of VEGF and other proangiogenic factors which stimulate the formation of new capillaries in the PM. In the pathogenesis of peritoneal fibrosis, peritoneal angiogenesis is known as the main mechanism besides peritoneal inflammation. The expanded vascular network increases effective surface area exchange and decreases the glucose-driven osmotic pressure and ultrafiltration. Several types of research also report the cooperation between vascularization and peritoneal fibrosis (Shi et al., 2020). Therefore, preventing or ameliorating peritoneal angiogenesis may serve as a potential therapeutic strategy for delaying peritoneal fibrosis and improving the efficiency of PD. In particular, a recent study has raised many attempts to investigate the molecular mechanisms involved in peritoneal angiogenesis, and a number of therapeutic strategies have been suggested to preserve the PM. In this study, we established a mouse peritoneal fibrosis model of PD by daily intraperitoneal injection with standard PD solution. After PD treatment, the pathological of the mouse peritoneum showed fibrosis characteristics. Meanwhile, peritoneum VEGF/VEGFR signaling was activated, and the level of CD31 and microvessel density of peritoneum increased significantly. The evidence proved that peritoneal angiogenesis arose in the development of peritoneal fibrosis. However, TMP treatment inhibited peritoneal angiogenesis, including partially attenuating the level of CD31 and microvessel density of the peritoneum. Peritoneal thickening and collagen deposition were also ameliorated after TMP treatment. Then, we demonstrated that VEGF secreted from HMrSV5 could stimulate HPVECs to activate VEGFR, and TMP treatment inhibited proliferation, migration, and tube formation of HPVECs cocultured with HMrSV5.

VEGF is recognized as a strong angiogenesis inducer and implicated in peritoneal membrane remodeling that limits ultrafiltration in PD. Knowledge of the mechanisms by which its ligand VEGFR executes diverse functions is of paramount importance. Accumulated evidence indicated that Hippo/YAP signaling has crosstalk with VEGF/VEGFR signaling in the process of angiogenesis (Zhu et al., 2018). YAP is closely related to angiogenesis. It can translocate to the nucleus and bind with the TEAD family, regulating its downstream signaling molecules such as CTGF, CYR61, and other proangiogenic factors. For example, CTGF and CYR61 are members of the CCN family, which are a group of secreted multifunctional proteins interacting with the extracellular matrix and associated with adhesion, migration, and mitogenesis (Brigstock, 2002; Toda et al., 2018). Thus, blocking the nuclear translocation and activation of YAP are beneficial for antagonizing angiogenesis. In this study, VEGF induced angiogenesis *via* Hippo/YAP signaling, characterized by increasing levels of Lats, YAP, CTGF, and CYR61. Our results indicated that TMP suppressed angiogenesis by upregulating YAP phosphorylation and downregulating YAP nuclear translocation. Meanwhile, YAP knockdown inhibited migration and tube formation of HPVECs. In a mouse model of PD, YAP knockdown inhibited the level of CD31 and microvessel density of the peritoneum and also ameliorated peritoneal fibrosis. These studies suggested that YAP was a key point in remission of peritoneal angiogenesis.

It is crucial to demonstrate the effect of TMP on YAP. As an important transcription coactivator, YAP also regulates the expressions of several cytoskeletal remodeling genes, including the gene encoding myosin 1C. It was reported that cell surface VEGFR exists in a dynamic state and its levels are maintained by internalization, recycling, secretory transport, and delivery of receptor to the plasma membrane. The cell surface distribution of VEGFR was regulated by myosin 1C in endothelial cells. Additionally, the Golgi apparatus located in the perinuclear region is known as a “post station”. It receives newly synthesized proteins and lipids from the endoplasmic reticulum, sequentially modifies, and dispatches them to distinct destinations by protein sorting at the trans-Golgi network (TGN) (Klute et al., 2011; Brandizzi and Barlowe, 2013). Much of the recent efforts to define the mechanism of Golgi structure and function have been put on Golgi structural proteins including Golgins and GRASPs, actin and cytoskeletons, and other GTPases (Prydz et al., 2020). Interestingly, myosin 1C also regulated the Golgi structure and function. In fact, in the present study, we used western blotting to confirm the increased distribution of VEGFR at the cell surface in HPVECs with YAP overexpression, which was alleviated by TMP treatment. Immunofluorescence assay showed that a fraction of VEGFR in the perinuclear region colocalized with TGN46 in HPVECs, indicating that VEGFR was present in Golgi. YAP overexpression led to reduced colocalization, but the change was reversed by TMP treatment. So it was reasonable that TMP exerted its antiangiogenesis effect by blocking YAP-induced VEGFR trafficking.

VEGFR is expressed at the endothelial cell surface and regulates angiogenesis in response to VEGF. Binding of VEGF to VEGFR2 at the cell surface initiates signal transduction such as the ERK/MAPK pathway being involved in the proliferation of endothelial cells (Giurdanella et al., 2020); PI3K/Akt pathway being involved in endothelial cell survival, vasodilatation, and vascular permeability (Wang et al., 2020); and p38 MAPK pathway weakening the intercellular junctions, destabilizing the cytoskeleton of endothelial cells, and inducing the formation of endothelial fenestrae (Cébe et al., 2006). As mentioned before, VEGFR from Golgi for delivery to the plasma membrane was involved in the distribution of VEGFR at the cell surface in HPVECs. VEGFR at the cell surface was remarkably increased in HPVECs with YAP overexpression, and much more VEGFR would be activated by VEGF stimulation under the circumstances. Consistently, western blotting revealed that the downstream signaling events of VEGFR, exemplified by p-ERK, p-P38, and p-Akt, were increased significantly in HPVECs with YAP overexpression. Therefore, we confirmed that a positive feedback loop was established during peritoneal angiogenesis. YAP-regulated VEGF induced signal transduction by enhanced VEGFR localization at HPVECs cell surface.

In this study, the most important finding is a validation of antiangiogenesis effect of TMP in peritoneal vascular endothelial cells through the regulation of YAP activation. TMP inhibits YAP nuclear translocation and attenuates VEGFR trafficking from Golgi to the cell surface, which may further inhibit YAP-dependent positive feedback loop. These mechanisms highlight the role of TMP in the regulatory effect of YAP and its vital function during peritoneal angiogenesis.

## Conclusion

In summary, our study illustrated the inhibitory effect of TMP on peritoneal angiogenesis *in vitro* and *in vivo*. The effect of TMP on peritoneal angiogenesis was demonstrated to be regulated by VEGF/Hippo/YAP signaling. TMP inhibits the nuclear translocation of YAP and attenuates VEGFR trafficking from Golgi to the cell surface, which may further inhibit YAP-dependent positive feedback loop. Our findings reveal a potential therapeutic use of TMP in the prevention or treatment of peritoneal fibrosis.

## Data Availability

The original contributions presented in the study are included in the article/Supplementary Material; further inquiries can be directed to the corresponding author.
